# Five new species of the genus *Primulina* (Gesneriaceae) from Limestone Areas of Guangxi Zhuangzu Autonomous Region, China

**DOI:** 10.3897/phytokeys.127.35445

**Published:** 2019-07-19

**Authors:** Shu Li, Zi-Bing Xin, Wei-Chuen Chou, Yi Huang, Bo Pan, Stephen Maciejewski, Fang Wen

**Affiliations:** 1 Guangxi Key Laboratory of Plant Conservation and Restoration Ecology in Karst Terrain, Guangxi Institute of Botany, CAS & Guangxi Zhuangzu Autonomous Region and Chinese Academy of Sciences, Guilin 541006, China; 2 Gesneriad Conservation Center of China (GCCC), Guilin Botanical Garden, CAS, Guilin 541006, China; 3 Key Laboratory of Plant Resource Conservation and Sustainable Utilization, South China Botanical Garden, Chinese Academy of Sciences, Guangzhou 510650, China; 4 The Gesneriad Society, 2030 Fitzwater Street, Philadelphia, PA 19146, USA

**Keywords:** Biodiversity, flora of Guangxi, karst, lithophilic plant, new taxon, taxonomy

## Abstract

Five new species of *Primulina* (Gesneriaceae) are described and illustrated here, namely *P.purpureokylin* F. Wen, Yi Huang & W. Chuen Chou, *P.persica* F. Wen, Yi Huang & W. Chuen Chou, *P.cerina* F. Wen, Yi Huang & W. Chuen Chou, *P.niveolanosa* F. Wen, S. Li & W. Chuen Chou and *P.leiyyi* F. Wen, Z.B. Xin & W. Chuen Chou. The characteristic traits of these species, together with photographs, detailed descriptions, notes on etymology, distribution, and habitat, as well as comparisons with morphologically similar species, are provided.

## Introduction

The genus *Primulinasensu lato*, as a group, is representative of the rich diversity of Chinese Gesneriaceae. The tropical and subtropical karst limestone mountainous areas of Guangxi, China, are the centers of species diversity and differentiation of this genus. As of the end of June 2017, the accepted and published species of *Primulina* from China had already reached 180 (includinginfraspecific taxa). Among them, 115 species (more than 63%) are confirmed and recorded from Guangxi Zhuangzu Autonomous Region (Guangxi for short) (Xu et al. 2017). Recently, an additional 16 new taxa of *Primulina* from China were published. By the end of January 2019, at least 196 species of *Primulina* were recorded from China (Wen et al. 2019). Many new taxa of *Primulina* from South and Southwest China are being discovered and published. After reviewing the genus *Primulina* and collating the new taxa for about two years (2017–2018), we discovered that there were a total of 11 new taxa from Guangxi. They are *P.lutescens* B. Pan & H. S. Ma (Ma et al. 2017), *P.albicalyx* B. Pan & Li H. Yang (Yang and Pan 2017), *P.curvituba* B. Pan, L.H. Yang & M. Kang (Yang et al. 2017), *P.dichroantha* F. Wen, Y.G. Wei & S.B. Zhou (Wu et al. 2017), *P.yandongensis* Ying Qin & Yan Liu (Qin et al. 2018), *P.hiemalis* Xin Hong & F. Wen, *P.davidioides* F. Wen & Xin Hong (Hong et al. 2018b), *P.zhoui* F. Wen & Z.B. Xin, *P.huangii* F. Wen & Z.B. Xin (Xin et al. 2018), *P.cangwuensis* X. Hong & F. Wen (Hong et al. 2018a), P.hochiensis(C.C. Huang & X.X. Chen)Mich. Möller & A. Webervar.ovata L.H. Yang, H.H. Kong & M. Kang (Yang et al. 2019). Guangxi is located in southern China. Most of the province originates from erosion of a limestone plateau and has a subtropical monsoon-affected climate. Several new species of Gesneriaceae have been discovered and published in recent years from this region. We suspect that more new taxa of *Primulina*, and even Gesneriaceae, are still to be found here.

The Guangxi Institute of Botany continues to support the Gesneriad Conservation Center of China (GCCC) and the Guilin Botanical Garden, CAS, in carrying out the investigations of Gesneriaceae diversity in S & SW China in recent years. Since 2015, the GCCC has been introducing, conserving, and propagating a large number of *Primulina* plants from S and SW China and N Vietnam. Many *Primulina* species from the Guangxi area are new to science, and waiting to be further studied. After careful study of both relevant specimens and taxonomic publications from the adjacent regions (Wang et al. 1990, 1998; Li and Wang 2005, Wei et al. 2010; Weber et al. 2011; Möller et al. 2016; Xu et al. 2017), we concluded that these plants represent five new species of *Primulina*. Descriptions, figures, and photos of these plants are presented here, as are morphological characters which are compared with those of closely related species.

## Taxonomic treatment

### Primulina purpureokylin

Taxon classificationPlantaeLamialesGesneriaceae

F.Wen, Yi Huang & W.Chuen Chou sp. nov.

urn:lsid:ipni.org:names:77199643-1

Fig. 1

#### Diagnosis.

*Primulinapurpureokylin* most resembles *P.leprosa* (Yan Liu & W.B. Xu) W.B. Xu & K.F. Chung (Fig. 6 A) (Xu et al. 2010, 2012) in having similarly purple indumentum on both surfaces of the leaf blade, but differs in having fewer leaves (4–6 in *P.purpureokylin* vs 5–12 in *P.leprosa*; same order as following), smaller leaf blade size (2–6.5 × 1.5–3.5 cm vs. 6–13 × 4–8 cm), bracts shape (linear or linear-lanceolate vs broadly ovate), corolla color (pinkish purple vs. yellow) and length (1.5–1.8 cm long vs. ca. 2.3 cm long), staminodes number (2 vs 3) and indumentum of style (nearly glabrous vs glandular-pubescent).

#### Type.

CHINA. Pingguo County, Xin’an Town, Gusha village, 23°16'N, 107°29'E, 200 m a.s.l., growing on the surface and crevices of moist limestone rocks at the bottom of cliffs, 3 Apr 2018, *Chou Wei Chuen et al. CWC171116-01* (holotype: IBK!, isotype: IBK!).

#### Description.

Perennial herbs. Rhizome subterete, 1–1.5 cm long, 5–10 mm in diameter. Leaves 4–6, all basal, opposite pairs; petiole compressed, cross section semi-elliptic, 1.5–2.5 cm long, 0.6–1.1 cm wide, shortly reddish purple to purplish brown strigose on both surfaces; blades dark green to purplish green, coriaceous or stiffly chartaceous, obliquely ovate, elliptic to broadly oblong-ovate, left-right asymmetric or symmetric, 2–6.5 × 1.5–3.5 cm, upper surface distinctly bullate, cuneate at base, commonly symmetric, occasionally oblique, margin entire, obtuse or rounded at apex, with erect reddish purple pubescence on both surfaces, margins with ciliate, pubescence 0.5–1 mm long, lateral veins 3 or 4 on each side, impressed adaxially and prominent abaxially. Cymes axillary, 2–4, 1- or 2-branched or single, 1- or 2–4-flowered on one cyme; peduncle 5.5–14.5 cm long, slender, 1–1.2 mm in diam., erect white glandular-pubescent; bracts 2, opposite, purplish green, linear or linear-lanceolate, ca. 6 × 1.5 mm, margin entire, acute at apex, sparsely purple puberulent outside, glabrous inside. Bracteoles 2, opposite, shape, hairs and color same as bracts but obviously smaller, ca. 3 × 0.8 mm. Pedicel 1–1.6 cm long, 0.8–1 mm in diam. Calyx 5-parted near to the base, lobes narrowly lanceolate-linear, 3–4 × 0.8–1 mm, margin entire, acute at apex, spreading white pubescent outside, glabrous nearly inside. Corolla pinkish purple, within 8–10 longitudinal dark purple stripes from corolla throat to the bottom of corolla tube, 1.5–1.8 cm long, spreading glandular puberulent outside, glabrous inside; tube tubular, pink, 8–9 mm long, 5–6 mm in diameter in medium, 5.5–6.5 mm in diam. at the mouth; limb distinctly 2-lipped, dark pink to purplish pink, adaxial lip 2-lobed to the middle, lobes ovate or nearly oblong, ca. 5 × 3 mm, obtuse to rounded at apex, with 2 or 3 deep purple lines inside; abaxial lip 3-lobed over the middle, two lateral lobes in apparently obliquely oblong, the central one zygomorphic, oblong, ca. 7 × 4 mm, rounded at apex. Stamens 2, adnate to ca. 5.5 mm above the base of the corolla tube; filaments white, geniculate at the middle, ca. 5.5 mm long, glabrous; anthers pale brown to purplish brown, subreniform, slightly contracted in the middle, ca. 1 mm long, glabrous. Staminodes 2, translucent, 0.8–1 mm long, glabrous, adnate to ca. 5 mm above the base of the corolla tube. Disc annular, ca. 0.7 mm high, margin entire. Pistil 1–1.1 cm long, ovary yellowish brown, linear, ca. 5 mm long, 0.8–1 mm in diam., glandular-puberulent; style white to translucent, 5–6 mm long, nearly glabrous; stigma obtrapeziform, ca. 0.9 mm long, apex 2-lobed. Capsule glabrous, valvate dehiscence when mature, 1.5–2 cm long.

**Figure 1.**
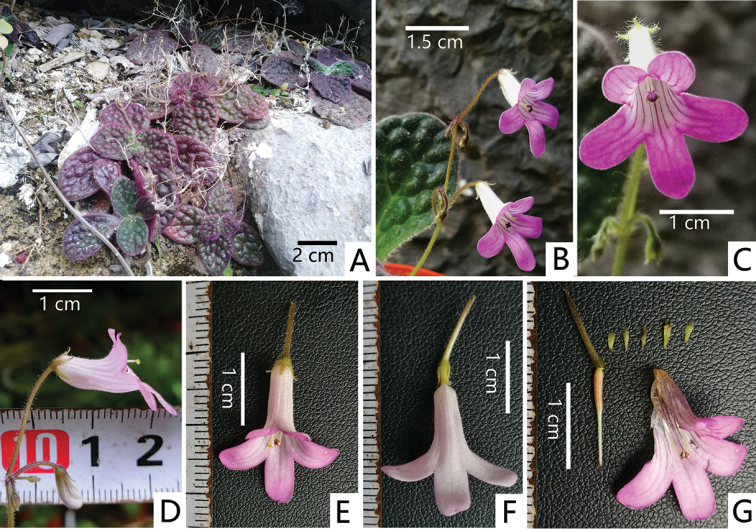
*Primulinapurpureokylin* F.Wen, Yi Huang & W.Chuen Chou sp. nov. **A** habit **B** cyme **C** frontal view of corolla **D** lateral view of corolla **E** dorsal view of corolla **F** ventral view of corolla **G** opened corolla for showing stamens, with pistil and calyx. Photographed by Wei-Chuen Chou and Yi Huang, charted by Wen-Hua Xu.

#### Phenology.

Flowering in Nov., fruiting time in Dec.

#### Etymology.

The specific epithet, ‘*purpureokylin*’, consists of two parts. The first part of the scientific epithet is “*purpure*-“, means purple. It refers to the upper faces of the leaf blades which are covered in purple pubescent-hairs. The second half of the epithet, “*kylin*”, refers to one of the auspicious animals in the traditional culture of China. Because the interesting and beautiful leaves are full of purple-hairs, bubbles on the surface appear as if covered with the purple squamae of Kylin. The Chinese name is “Zí Líng Bào Chūn Jù Tái” (紫麟报春苣苔).

#### Distribution and habitat.

*Primulinapurpureokylin* is currently known only from the type locality. Only a single population with ca. 100 individuals was discovered and confirmed. The species is only known growing on the surface and crevices of wet limestone rocks at the bottom of cliffs in Pingguo County, Guangxi, China.

#### Provisional IUCN conservation assessment.

Along with the further field investigations for the current survival situation of *Primulinapurpureokylin*, the extinction risk of this species is rising because of over-harvesting by local plant collectors. Overexploited because of its beauty, this unpublished species is on the brink of extinction. Although more surveys are needed to clarify its conservation status, the provisional conservation status is Critically Endangered CR B2ab (iii, v) according to the IUCN red list criteria (IUCN 2012).

### Primulina persica

Taxon classificationPlantaeLamialesGesneriaceae

F.Wen, Yi Huang & W.Chuen Chou sp. nov.

urn:lsid:ipni.org:names:77199644-1

Fig. 2

#### Diagnosis.

*Primulinapersica* most closely resembles *P.gongchengensis* Y.S. Huang & Yan Liu (Fig. 6 B) (Huang et al. 2012) in having similarly shaped leaf blades. It differs from the latter by having a different indumentum on both surfaces of the leaf blades (densely eglandular-pubescent in *P.persica* vs densely glandular-pubescent in *P.gongchengensis*; same order as following), margin of leaf blade (irregularly serrate in different numbers vs. repand or crenate), smaller bracts (6–7 × 1.5–2 mm vs. 10–20 × 2–3.5 mm) and bracts shape (linear vs. narrowly rhombic to oblong), the indumentum of calyx lobes inside (glabrous vs sparsely glandular-pubescent) and shorter corolla length (7.5–10 mm long vs. 22–28 mm long).

#### Type.

CHINA. Yangshuo County, Gaotian Town, Lexiang village, 24°42'N, 110°30'E, 124 m a.s.l., growing on the surface of tufa and crevices of moist rocks on a cliff of a limestone hill, 3 Apr 2018, *Chou Wei Chuen et al. CWC171116-01* (holotype: IBK!, isotypes: IBK!)

#### Description.

Perennial herbs. Rhizome subterete, 5–6 cm long, 8–10 mm in diam. Leaves 6–10, crowded at apex of rhizome, petiolate; petiole cylindrical, upper slightly smaller and the bottom slightly inflated but the base slightly applanate, 8–15 cm long, 6–8 mm in diam. at base, densely eglandular-pubescent; leaf blade herbaceous, rhomboid-ovate or elliptic, 6–11 × 5–9.5 cm, apex acute, obtuse or slightly round, base cordate, broadly cuneate to cordate, slightly inequilateral, margin irregularly serrate in different numbers, densely pubescent on both surfaces, lateral veins 2 or 3 on each side, impressed adaxially and prominent abaxially. Cymes 4–10, 1–3-branched, 12–30-flowered; peduncle 12–20 cm long, 2–2.5 mm across, densely glandular-pubescent; pedicel 1–3 cm long, densely glandular-pubescent; bracts opposite, linear, 6–7 × 1.5–2 mm, margin entire, apex acute, adaxially glandular-puberulent, abaxially nearly glabrous. Calyx 5-parted nearly to base, lobes narrowly lanceolate, 4.5–5.5 × ca. 1 mm, margin entire and ciliolate, outside densely glandular-pubescent, inside glabrous. Corolla pinkish, fuchsia to pale purple, 7.5–10 mm long, 4–4.5 mm in diam. at mouth, outside glandular-puberulent; tube short, 5.5–6.5 mm long, ca. 4.5 mm in diam. in middle, tube base slightly swollen, ca. 2 mm in diam. at base; limb distinctly 2-lipped, adaxial lip 2-lobed to more than the middle, lobes oblong, apex round, 3.2–3.8 × 2.5–3 mm, abaxial lip 3-lobed to more than the middle, lobes oblong, apex round, 4.5–5.2 × 3.3–3.6 mm. Stamens 2, adnate to 2.5 mm above corolla tube base, filaments 4–4.5 mm long, curved at middle, anthers elliptic or reniform, ca. 1.5 mm long, glabrous; staminodes 3, lateral ones 0.8–1 mm long, adnate to 1.8–2 mm above corolla tube base; middle one 0.5 mm long, adnate to ca. 1.3 mm above corolla tube base. Disc annular, ca. 0.6 mm in height, margin entire. Pistil 6.5–7.5 mm long, ovary ovoid, ca. 2.5 × 1.3–1.5 mm, glandular-puberulent; style indumentum same as ovary, 4–5 mm long, ca. 0.5 mm in diam. in the middle; stigma obtrapeziform, ca. 0.8 mm long, apex 2-lobed. Capsule ovoid, ca. 8 mm long, valvate dehiscence when mature, outside pubescent.

**Figure 2.**
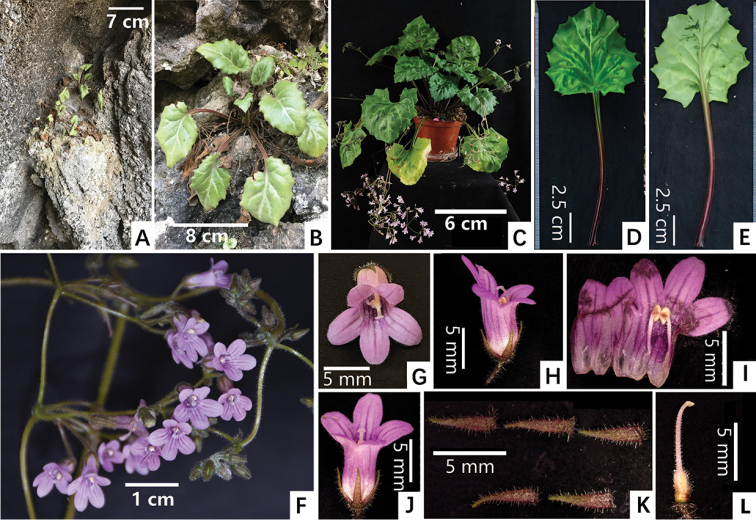
*Primulinapersica* F.Wen, Yi Huang & W.Chuen Chou sp. nov. **A** habitat **B** habit **C** plant in flower, cultivated in GCCC**D** adaxial surface of leaf **E** abaxial surface of leaf **F** cyme and flowers **G** front view of corolla **H** lateral view of corolla **I** opened corolla for showing stamens **J** dorsal view of corolla **K** adaxial surface of calyx lobes **L** pistil. Photographed by Fang Wen, charted by Wen-Hua Xu.

#### Phenology.

Flowering from May to June; fruiting from June to August.

#### Etymology.

The specific epithet, ‘*persica*’, refers to the color of its flower, a vivid and bright peach. The Chinese name is “Tāo Hóng Xiáo Huā Jù Tái” (桃红小花苣苔).

#### Distribution and habitat.

*Primulinapersica* is currently known only from the type locality and only about 50 individuals were confirmed. All individuals are growing on the surface of tufa and wet crevices of moist rocks on the cliff of a limestone hill in Yangshuo, Guangxi, China. There are no accompanying plants except for some ferns.

#### Provisional IUCN conservation assessment.

The original habitat of this species was almost destroyed because of road building in 2013; it directly resulted in a single small population with no more than 50 surviving individuals. We hope we can find more populations in the future through field surveys. The habitat of *Primulinapersica* is likely to be subjected to human activities because the survival population grows in cracks of a limestone cliff by the side of the road. Thus, based on currently available information, *P.persica* should be considered as ‘Critically Endangered’ (CR): B1+2ab(V); C2b, following the IUCN categories and criteria (IUCN 2012).

### Primulina cerina

Taxon classificationPlantaeLamialesGesneriaceae

F.Wen, Yi Huang & W.Chuen Chou sp. nov.

urn:lsid:ipni.org:names:77199645-1

Fig. 3

#### Diagnosis.

*Primulinacerina* most closely resembles *P.renifolia* (D. Fang & D.H. Qin) J.M. Li & Y.Z. Wang (Fig. 6 C) (Fang and Qin 2004, Wang et al. 2011, Weber et al. 2011) in having similarly shaped leaf blades, but differs in their indumentum of the peduncle (densely erectly eglandular-puberulent in *P.cerina* vs spreading white pubescent and glandular puberulent in *P.renifolia*; same order as following), corolla tube shape (tubular, abaxially straight and not swollen vs. obliquely campanulate, abaxially swollen), corolla color (beige to pale yellow mixed slightly reddish brown vs. pale purple to purple inside longitudinally purple lines), indumentum of filaments (glabrous vs glabrous but base glandular puberulent), the indumentum of calyx lobes inside (glabrous vs sparsely glandular-pubescent) and stigma shape (obtrapeziform and 2-lobed vs obliquely hippocrepiform but unlobed).

#### Type.

CHINA. Yizhou city, Beiya Town, Xiaozhudong village, 24°22'N, 108°23'E, 220 m a.s.l., only known from crevices of moist rock surfaces at the entrances of a big limestone cave, 3 Apr 2018, *Chou Wei Chuen et al. CWC171116-01* (holotype: IBK!, isotypes: IBK!)

#### Description.

Perennial herbs. Rhizome small and short, indistinctive but nearly cylindrical, 3–8 mm long, 2–3 mm in diam. Leaves numerous, 8–20 or more, all basal, petiolate; petiole pale brownish purple to dark brownish purple, cylindrical, 7.5–12 cm long, 3–3.2 mm in diam., extremely short puberulent to nearly glabrous; leaf blade dark green, slightly fleshy to thickly chartaceous, herbaceous when dried, nearly rounded to cordate rounded, 3–5.5 × 3.5–5.5 cm, apex obtuse to rounded, base cordate to deeply cordate, margin undulant to crenate; lateral veins 3–4 on each side, slightly impressed adaxially and apparently prominent abaxially. Cymes 8–16, 1–3-branched, 16–30-flowered per cyme; peduncle 8–15 cm long, ca. 2.5 mm in diam., densely erectly eglandular-puberulent; pedicel 5–10 mm long, ca. 1 mm in diam., indumentum same as pedicel; bracts 2, opposite, brownish purple, linear to oblanceolate, 5–12 × 1–4 mm, margin entire to inconspicuously dentate, apex acuminate to acute, adaxially very shortly puberulent, abaxially nearly glabrous. Calyx 5-parted nearly to base, lobes brownish purple, narrowly lanceolate, 3–4 × ca. 1 mm, margin entire, outside sparsely extremely short puberulent, inside nearly glabrous. Corolla beige to pale yellow mixed slightly reddish brown, 1.8–2 cm long, 4–5 mm in diam. at mouth, outside sparsely eglandular-puberulent to nearly glabrous, inside glabrous; tube tubular, 1.5–1.8 cm long, 3.5–4 mm in diam. at the middle, tube base slightly constricted, 1–1.5 mm in diam. at base; limb distinctly 2-lipped, adaxial lip 2-lobed to base, lobes oblong, apex rounded, 2.5–3 × 2–2.5 mm, abaxial lip 3-lobed to over middle, lobes oblong, apex rounded, ca. 3.5 × 3 mm. Stamens 2, adnate to ca. 3 mm above corolla tube base, filaments ca. 6 mm long, curved at middle, anthers elliptic or reniform, ca. 1.8 mm long, glabrous; staminodes 2, extremely small and inconspicuous, punctate, ca. 0.05 mm long, adnate to corolla tube base. Disc annular, ca. 0.9 mm in height, margin entire to sinuate. Pistil 10.5–11 mm long, ovary ovoid, ca. 2.5 × 1 mm, densely eglandular-puberulent; style ca. 8 mm long, ca. 0.2 mm in diam. at the middle, the lower part of style sparsely eglandular- and glandular-puberulent but the upper half part of style nearly glabrous; stigma obtrapeziform, ca. 0.5 mm long, apex 2-lobed. Capsule ovoid, valvate dehiscence when mature, 4–4.5 × ca. 3 mm.

**Figure 3.**
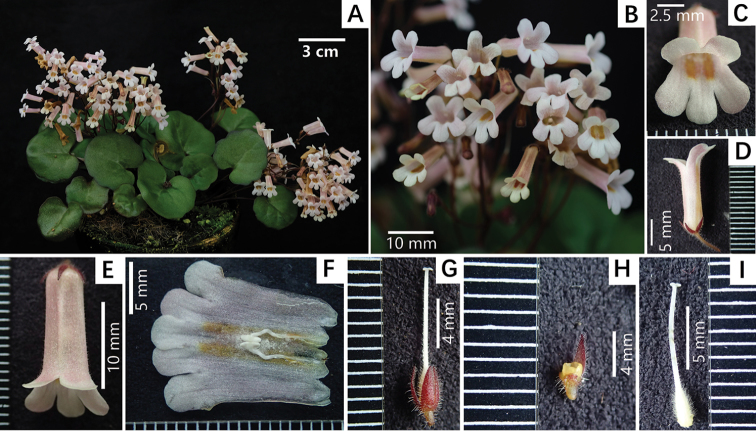
*Primulinacerina* F.Wen, Yi Huang & W.Chuen Chou sp. nov. **A** plant in flower, cultivated in GCCC**B** cyme with flowers **C** front view of corolla **D** lateral view of corolla **E** dorsal view of corolla **F** opened corolla for showing stamens **G** pistil and calyx lobes for showing their adaxial surfaces **H** disc and one of calyx lobes for showing its abaxial surface **I** pistil without calyx lobes for showing ovary and style and their indumentum. Photographed by Fang Wen, charted by Wen-Hua Xu.

#### Phenology.

Flowering from April to May; fruiting from June to July.

#### Etymology.

The specific epithet, ‘*cerina*’, refers to the special color of the flowers; ‘*cerina*’ is derived from the Latin, ‘*cerinus*’, meaning dark yellow or sulfur yellow, but mixed with a little pale reddish brown. The color seems like the hue of the natural brimstone (sulphur) ore. The Chinese name is “Àn Líu Sè Xiáo Huā Jù Tái” (暗硫色小花苣苔).

#### Distribution and habitat.

*Primulinacerina* is currently known only from the type locality. The species grows in the crevices of rocks with wet surfaces at the entrances of a large limestone cave in Yizhou, Guangxi, China.

#### Provisional IUCN conservation assessment.

*Primulinacerina* is rarer than the species mentioned above, *P.persica*. At present, only a single population with ca. 20 individuals is known, counted and confirmed from 2016 to 2018. Although the type locality is in an outlying mountain area and surrounded by limestone forest, this species should be assessed as ‘Critically Endangered, CR B2a+C2a(i,ii)+D’, given the few known individuals and a single population based on the IUCN categories and criteria (IUCN 2012).

### Primulina niveolanosa

Taxon classificationPlantaeLamialesGesneriaceae

F.Wen, S. Li & W.Chuen Chou sp. nov.

urn:lsid:ipni.org:names:77199646-1

Fig. 4

#### Diagnosis.

*Primulinaniveolanosa* most closely resembles *P.repanda* (W.T. Wang) Y.Z. Wang (Fig. 6 D) (Wang 1981, Wang et al. 2011, Weber et al. 2011) in having similarly shaped leaf blades, but differs in their indumentum of the leaf blades (both surfaces spreading densely long white villous to lanate in *P.niveolanosa* vs. appressed puberulent to villous in *P.repanda*; same order as following), number of bracts (3, whorled vs. 2, opposite), shape of bracts (narrowly oblong to broadly oblanceolate vs. lanceolate-linear to subulate), shape of calyx lobes (linear to narrowly oblanceolate vs. narrowly triangular), corolla length (1.5–1.8 cm long vs. ca. 8 mm long).

#### Type.

CHINA. Yizhou city, Beiya Town, Jiucai village, 24°24'N, 108°24'E, 181 m a.s.l., growing on the surface of a moist cliff at the edge of a village, 3 Apr 2018, *Chou Wei Chuen et al. CWC171116-01* (holotype: IBK!, isotypes: IBK!)

#### Description.

Perennial herbs. Rhizome cylindrical, 3–15 mm long, 1.5–3 mm in diam. Leaves numerous, 16–40 or more, all basal, petiolate; petiole green, oblong-oblate, 4–8 cm long, 6–15 mm in diam., spreading appressed white pubescent to lanate; leaf blade pale green to green, fleshy to thickly chartaceous, herbaceous when dried, narrowly oblong, narrowly oblanceolate to oblong-elliptic, 5–10 × 2.5–4.5 cm, apex obtuse and occasionally rounded, base gradually attenuated to form petiole, margin entire, undulatet to crenate, both surfaces densely villous to lanate with long, spreading white hairs, lateral veins 4 or 5 on each side, slightly impressed adaxially and apparently prominent abaxially. Cymes axillary, 6–12 per plant, 2–4-branched, 16–36-flowered per cyme; peduncle 15–25 cm long, 2.5–3.5 mm in diam., green, densely white villous and lanose; bracts 3, whorled, brownish green, margin entire to undulate, apex acuminate to acute, adaxially white pubescent, abaxially nearly glabrous, lateral ones bigger, ca. 15 × 6 mm, narrowly oblong to broadly oblanceolate, central one smaller, ca. 15 × 3.5 mm; pedicel 1–1.5 cm long, ca. 1 mm in diam., indumentum same as pedicel; pedicel green, 1–2.5 cm long, 1–1.5 mm in diam., densely white pubescent. Calyx 5-parted nearly to base, lobes green to brownish green, linear to narrowly oblanceolate, 5–7 × ca. 2 mm, margin entire, outside white puberulent, inside nearly glabrous. Corolla pale pink to white, inside with two dark pink to pinkish orange stripes, 1.5–1.8 cm long, 5–7.5 mm in diam. at mouth, outside and inside glabrous; tube infundibular, pale pink, 1.1–1.3 cm long, 4.5–5.5 mm in diam. at the middle, 1.3–1.8 mm in diam. at base; limb distinctly 2-lipped, adaxial lip 2-lobed to over middle, lobes oblong to rounded, apex rounded, 3.5–4 × ca. 4 mm, abaxial lip 3-lobed to over middle, lobes oblong to rounded, apex rounded, 4–4.5 × ca. 4 mm. Stamens 2, adnate to ca. 1.8–2.2 mm above corolla tube base, filaments 1.8–2.3 mm long, geniculate at 1/3 from the bottom, glabrous, anthers elliptic or reniform, ca. 1 mm long, ca.0.5 mm in diam. per anther, white beard; staminodes 3, glabrous, lateral ones adnate to 1.7–2 mm above corolla tube base, white to translucent, ca. 2 mm long, apex capitate, central one adnate to ca. 1.6 mm above corolla tube base, capitate. Disc annular, glabrous, ca.0.6 mm in height, margin entire to sinuate. Pistil ca. 8 mm long, ovary ovoid, ca. 2 × 1.3 mm, densely eglandular lanose; style ca. 6 mm long, ca. 0.3 mm in diam. at the middle, the lower part of style sparsely eglandular-puberulent but the upper half part of style nearly glabrous; stigma obtrapeziform, ca. 0.5 mm long, apex 2-lobed. Capsule ovoid, glabrous, valvate dehiscence when mature, 4–4.5 × ca. 3 mm.

**Figure 4.**
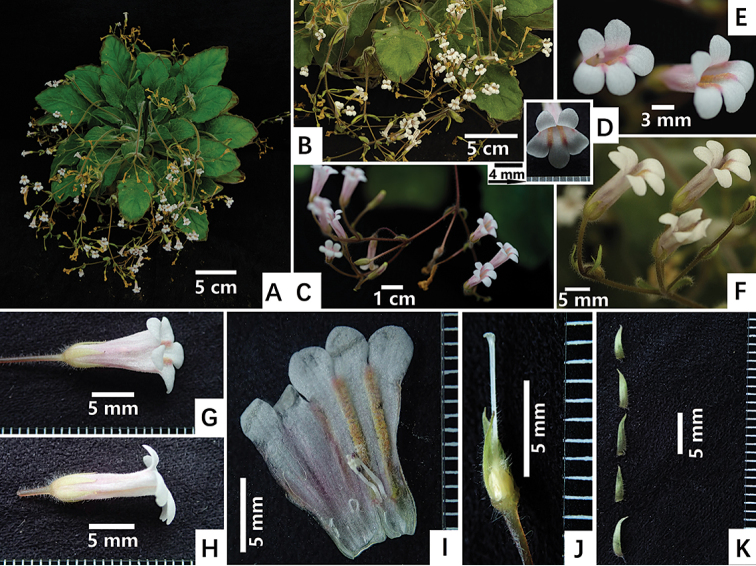
*Primulinaniveolanosa* F.Wen, S. Li & W.Chuen sp. nov. **A** plant in flower, cultivated in GCCC**B** cyme with flowers **C** close look of cyme and flowers **D** front view of corolla **E** lateral view of corolla **F** lateral view of corolla, bracts and bracteoles **G** dorsal view of corolla **H** lateral view of corolla with scale **I** opened corolla for showing stamens and staminodes **J** pistil with two calyx lobes for showing ovary and style and their indumentum **K** adaxial surface of calyx lobes. Photographed by Fang Wen, charted by Wen-Hua Xu.

#### Phenology.

Flowering from March to April; fruiting from April to May.

#### Etymology.

The specific epithet, ‘*niveolanosa*’, consists of two parts. The first part of “*niveo*”, comes from the Latin word, ‘*niveus*’, and means as white as snow, or snow-white; the second half, ‘*lanosa*’, is from the Latin, ‘*lanosus*, *lani*-’, meaning with lanose or villous hairs. Thus, the scientific name refers to the plants seemingly covered with snow because of the indumentum of snowy lanose hairs. The Chinese name is “Mián Máo Xiáo Huā Jù Tái” (绵毛小花苣苔).

#### Distribution and habitat.

*Primulinaniveolanosa* is currently known only from the type locality: a single population with ca. 100 individuals, and although it might be endangered, more surveys are needed to clarify its conservation status. The species is only known growing on the surface of a moist cliff along the edge of a village.

#### Provisional IUCN conservation assessment.

About 100 mature individuals have been recorded and confirmed, growing on a wet rock surface under evergreen broad-leaved forest on a limestone hill. Because the local government of Yizhou city is planning to develop a scenic spot project at this place, the habitat of this new species is likely to be subjected to human activities. Thus, based on currently available information, *P.niveolanosa* should be considered as ‘Critically Endangered’ (CR): B1+2ab(V); C2b, following the IUCN categories and criteria (IUCN 2012).

### Primulina leiyyi

Taxon classificationPlantaeLamialesGesneriaceae

F.Wen, Z.B. Xin & W.Chuen Chou sp. nov.

urn:lsid:ipni.org:names:77199647-1

Fig. 5

#### Diagnosis.

*Primulinaleiyyi* most closely resembles *P.longgangensis* (W.T. Wang) Yan Liu & Y.Z. Wang (Fig. 6 E) (Wang and Huang 1982, Wang et al. 2011, Weber et al. 2011) in having similarly shaped leaf blades, but differs in their indumentum of young stems (sparsely appressed hazel pubescent in *P.leiyyi* vs. nearly glabrous in *P.longgangensis*; same order as following), shape of bracts (elliptic to cymbiform vs. linear to lanceolate or obovate), indumentum of corolla (outside sparsely glandular-puberulent, inside glabrous vs. outside glabrous to puberulent, inside puberulent or glabrous below stamens), pistil length (1.8–2.0 cm long vs. ca. 2.8 cm long) and capsule length (4.8–5.5 cm long vs. 1.6–2.5 cm long)

#### Type.

CHINA. Nanning city, Suxu Town, Shibaluohandong village, 22°32'N, 108°3'E, 150 m a.s.l., growing on the top of limestone cliff near road, 3 Apr 2018, *Lei YuYang et al. LYY181208-01* (holotype: IBK!, isotypes: IBK!)

#### Description.

Perennial herbs. Rhizome cylindrical, the long rhizome up to 50 cm long or longer and branched repeatedly after several years of growth, 5–6 mm in diam.; branch up to 8–15 cm long, sparsely appressed hazel pubescent when young, gradually glabrous when mature. Leaves ternate, occasionally opposite, subsessile to sessile; leaf blade fleshly to thickly herbaceous when fresh, hard chartaceous when dried, often asymmetric, obliquely oblanceolate to broadly ensiform, 6.5–16 × 1.5–2.5 cm, apex acuminate to acute, base narrowly cuneate, gradually attenuated to be petiole, margin entire, adaxially and abaxially densely appressed pubescent and strigose, lateral veins 4 or 5 on each side, slightly impressed adaxially and apparently prominent abaxially. Inflorescences axillary, cymes 2–6 on the near top of every branch, 1-branched, (1)2–6-flowered per cyme; peduncle reddish brown, 3–7.5 cm long, 1–1.5 mm in diam., densely spreading eglandular-pubescent; bracts 2, opposite, green to yellowish green, elliptic to cymbiform, 1.2–2.3 cm × 3–6.5 mm, margin entire, apex acuminate to acute, adaxially and abaxially densely appressed pubescent; pedicel reddish brown, 2–3 cm long, ca. 1 mm in diam., indumentum same as peduncle. Calyx 5-parted to base, lobes reddish brown, narrowly lanceolate-linear to linear, ca. 8 × 0.3 mm, margin entire, outside densely puberulent, inside nearly glabrous. Corolla dark pink to purplish pink, ca. 3.5 cm long, 11–12.5 mm in diam. at mouth, outside sparsely glandular-puberulent, inside glabrous; tube tubular, 2.4–2.8 cm long, 3–3.5 mm in diam. at the middle, tube slightly upswept, base gradually constricted, 2.5–3.5 mm in diam. at base; limb distinctly 2-lipped, adaxial lip 2-lobed to over middle, slightly obliquely oblong, apex rounded, 4.5–5.5 × 3.8–4.5 mm, abaxial lip 3-lobed to about middle, lateral ones slightly obliquely oblong, 6.5–7.5 × ca. 4.5 mm, central one oblong, apex rounded, 7.5–8 × 6.5–7 mm, mm. Stamens 2, adnate to ca. 7 mm above corolla tube base, filaments white, 8–9 mm long, geniculate at middle, anthers white, reniform to fusiform, ca. 3 mm long, nearly glabrous; staminodes 3, lateral translucent to white, ones adnate to 6.2–6.5 mm above corolla tube base, 4–4.5 mm long, apex capitate, central one adnate to 4.8–5 mm above corolla tube base, small, punctate, ca. 1 mm long. Disc annular, white, 0.8–1 mm in height, margin entire. Pistil 1.8–2.0 cm long, ovary reddish brown, cylindric, 9–10 mm long, 1–1.3 mm in diam., densely white glandular-puberulent; style 9–10 mm long, 0.6–0.7 mm in diam., pale reddish brown from case to middle, white from middle to top, densely glandular-puberulent; stigma obtrapeziform, ca. 1.2 mm long, apex 2-lobed, lobes truncate. Capsule linear, valvate dehiscence and glabrous when mature, 4.8–5.5 cm × 2.2–2.5 mm.

**Figure 5.**
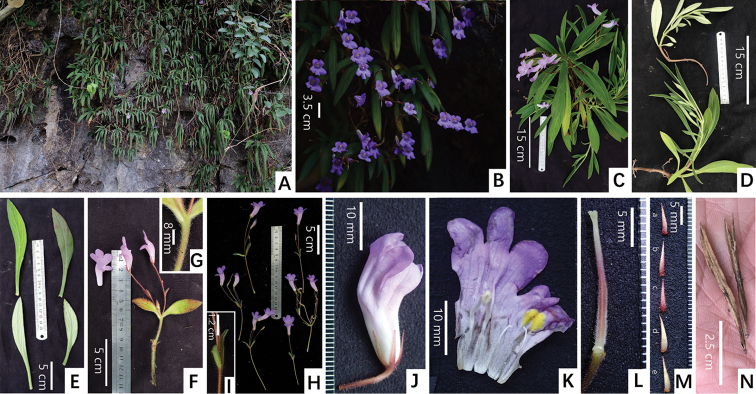
*Primulinaleiyyi* F.Wen, Z.B. Xin & W.Chuen sp. nov. **A** habit **B** cyme and flowers **C** plant in flower collected in the field **D** extended branches after growing for one or some years **E** adaxial and abaxial view of leaf blades **F** cyme with one flower near top of stem **G** pubescent hairs on the surface of stem **H** different cymes with variable numbers of flowers **I** bracts **J** lateral view of bud **K** opened corolla for showing stamens and staminodes **L** pistil without calyx lobes **M** adaxial surfaces of calyx lobes **N** mature capsules. Photographed by Fang Wen, charted by Wen-Hua Xu.

**Figure 6.**
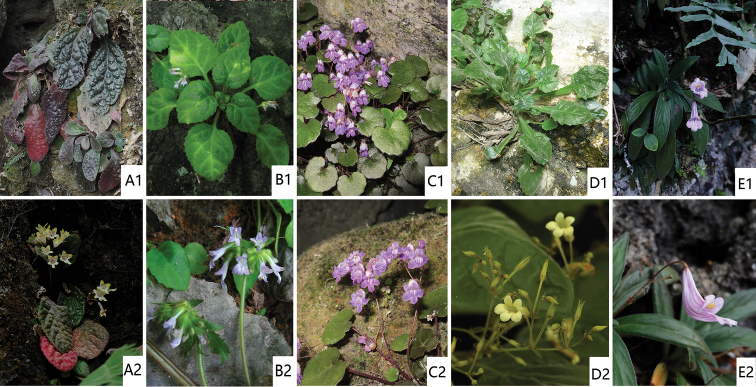
The morphologically similar congeners of the five new species of *Primulina***1** habit **2** flowers. **A***P.leprosa*, the congener of *P.purpureokylin***B***P.gongchengensis*, the congener of *P.persica***C***P.renifolia*, the congener of *P.cerina***D***P.repanda*, the congener of *P.niveolanosa***E***P.longgangensis*, the congener of *P.leiyyi.* Photographed by Fang Wen, charted by Wen-Hua Xu.

#### Phenology.

Flowering from November to the beginning of December; fruiting in January of the next year.

#### Etymology.

The new species is named after Mr. Yu-Yang Lei, who first discovered and collected this rare species and who accompanied us on a number of subsequent field expeditions in Nanning, Guangxi. The Chinese name is “Léi Shì Bào Chūn Jù Tái” (雷氏报春苣苔).

#### Distribution and habitat.

It is currently known only from the type locality in a single population with ca. 100 individuals. Although it might be endangered, more surveys are needed to clarify its conservation status. The species is only known growing on the surface of wet rocks on the limestone hills along the edges of village roads.

#### Provisional IUCN conservation assessment.

We carefully explored the type locality on five visits over a period of three years. *Primulinaleiyyi* appears to be restricted to limestone hills surrounding Shibaluohandong village, Suxu Town, Nanning city. Although the habitats are very near to densely populated areas and are easily threatened by human activities, this species is common and locally abundant on limestone hills. Using the IUCN Red List categories (IUCN 2012), a provisional conservation status of Least Concern (LC), is assessed for this species.

## Supplementary Material

XML Treatment for Primulina purpureokylin

XML Treatment for Primulina persica

XML Treatment for Primulina cerina

XML Treatment for Primulina niveolanosa

XML Treatment for Primulina leiyyi
